# UHPLC–MS/MS Analysis on Flavonoids Composition in *Astragalus membranaceus* and Their Antioxidant Activity

**DOI:** 10.3390/antiox10111852

**Published:** 2021-11-22

**Authors:** Zhili Sheng, Yueming Jiang, Junmei Liu, Bao Yang

**Affiliations:** 1 Guangdong Provincial Key Laboratory of Applied Botany, South China Botanical Garden, Chinese Academy of Sciences, Guangzhou 510650, China; shengzl@scbg.ac.cn (Z.S.); ymjiang@scbg.ac.cn (Y.J.); 2 College of Food Science and Engineering, Jilin Agricultural University, Changchun 130118, China; spring430817@163.com; 3 University of Chinese Academy of Sciences, Beijing 100049, China

**Keywords:** antioxidant activity, ORAC, origin, phenolics, principal component analysis

## Abstract

*Astragalus membranaceus* is a valuable medicinal plant species widely distributed in Asia. Its root is the main medicinal tissue rich in methoxylated flavonoids. Origin can highly influence the chemical composition and bioactivity. To characterize the principal chemicals influenced by origin and provide more information about their antioxidant profile, the extracts of *A. membranaceus* roots from four origins were analysed by UHPLC-MS/MS. Thirty-four flavonoids, including thirteen methoxylated flavonoids, fifteen flavonoid glycosides and six flavonols, were identified. By principal component analysis, eighteen identified compounds were considered to be principal compounds. They could be used to differentiate *A. membranaceus* from Shanxi, Inner Mongolia, Heilongjiang and Gansu. The antioxidant activity was analysed by ORAC assay, DPPH radical scavenging activity assay and cell antioxidant activity assay. ‘Inner Mongolia’ extract showed the highest antioxidant activity. These results were helpful to understand how origin influenced the quality of *A. membranaceus*.

## 1. Introduction

*Astragalus membranaceus (Fabaceae)* is a well-known traditional Chinese herbal medicine, which is mainly distributed in Asian regions [1]. The root of *A. membranaceus* is the medicinal tissue widely used due to their beneficial effects to lung health, which has been further proved to exhibit immunomodulatory, antioxidant, antiperspirant, antidiarrheal, and antidiabetic activities [2]. The bioactive compounds in the root of *A. membranaceus* are complicated. Previous research has revealed that the main bioactivities depend on the presence of non-volatile components, such as polysaccharides, saponins, and phenolics [3,4].

Flavonoids are a natural bioactive compound with C6-C3-C6 skeleton. They are commonly found in plant species, and possess good bioactivities which have been applied in nutraceutical, medicinal, and cosmetic products [5]. Many different subclasses of flavonoids have been described from *A. membranaceus* including flavone, flavonol, flavanone, flavanonol, chalcone, aurone, isoflavone, and pterocarpan [6]. Phenolics, including flavonoids, have been judged as the marker for quality evaluation and standardization of *A. membranaceus* and its processed products [7]. Methoxylated flavonoids and their glycosides, such as calycosin and calycosin-7-*O*-*β*-D-glucoside, were the major bioactive constituents of *A. membranaceus* due to their superior bioactivities [8,9]. After glycosylation and methylation, the reactivity and solubility of the flavonoids usually improved and thereby so did their absorption and bioactivity [10]. Many methoxyl flavones and flavonoid glycosides have been proved the superior antioxidant activities [11,12]. Due to the plant origin and extremely low toxicity, they have become the hotspots of natural antioxidants drug discovery and development [13,14].

The accumulation of bioactive compounds depends on geographical location and growing environment, including climate, soil, and fertilizer [15]. It is highly correlated to the health benefits and pharmaceutical activities. Different origins usually lead to different phytochemical profiles, which further relate to the quality of *A. membranaceus*. However, relevant information regarding this topic is limited. Therefore, in the present study, *A. membranaceus* from four production origins (Gansu, Shanxi, Inner Mongolia, and Heilongjiang provinces of China) were collected and the flavonoids were identified by ultra-high-performance liquid chromatography-tandem mass spectrometry (UHPLC-MS/MS). Afterward, the antioxidant activities were investigated by ORAC assay, DPPH radical scavenging activity assay and CAA assay. These results help to understand the qualities of *A. membranaceus* from different origins.

## 2. Materials and Methods

### 2.1. Plant Materials and Reagents

The dried root of *A. membranaceus* samples were obtained from Shanxi, Inner Mongolia, Gansu, and Heilongjiang provinces, respectively. They were screened for the consistency of shape, diameter, and length. The reagents used in the present work were of analytical grade. The methanol was purchased from Macklin Biochemical Co., Ltd. (Shanghai, China). Folin-Ciocalteu reagent, sodium carbonate (Na_2_CO_3_), aluminum chloride (AlCl_3_), sodium nitrite (NaNO_2_), sodium hydroxide (NaOH), potassium dihydrogen phosphate (KH_2_PO_4_), and dipotassium hydrogen phosphate (K_2_HPO_4_) were obtained from Sangon Biotech (Shanghai, China). Gallic acid, catechin, ascorbic acid, fluorescein sodium, trolox, 1,1-diphenyl-2-picryldydrazyl (DPPH), 3-(4,5-dimethylthiazol-2-yl)-5(3-carboxymethonyphenol)-2-(4-sulfophenyl)-2H-tetrazolium (MTS), glutamic acid, and 2,2′-azobis-amidinopropane (ABAP) were purchased from Sigma Chemical Co. (St. Louis, MO, USA). HPLC-grade methanol was obtained from Merck Co., Ltd. (Darmstadt, Germany). Dulbecco’s modified Eagle’s medium (DMEM), fetal bovine serum (FBS), and phosphate-buffered saline (PBS) for cell culture were purchased from Gibco Life Technologies (Grand Island, NY, USA). All the other chemicals were obtained from Tianjin Chemical Reagent Co. (Tianjin, China).

### 2.2. Extraction

The bioactive compounds were extracted as explained previously by Wu et al. [16] with some modifications. Five hundred milligrams of *A. membranaceus* root powder were extracted with 5 mL of methanol for one week in dark at room temperature. The extract was centrifuged at 9000× *g* for 20 min. Then the supernatants were collected and concentrated at 45 °C by a vacuum rotary evaporator (Eyela N-1100 V-W, Tokyo Ri-kakikai Co. Ltd., Tokyo, Japan). The aliquots were stored at 4 °C before use. The extraction was conducted in triplicate.

### 2.3. Determination of Total Phenolics and Flavonoids Contents

The content of total phenolics was determined by using Folin-Ciocalteu colorimetric protocol [17]. Briefly, an aliquot (1 mL) of the extract or standard solution of gallic acid (0, 20, 60, 100, 150, 200, 300, 400, 500, and 600 µg/mL) was mixed with 0.4 mL of distilled water and incubated for 6 min with 0.1 mL of Folin-Ciocalteu phenol reagent. Then 1.0 mL of Na_2_CO_3_ (7%, *w/v*) and 0.8 mL of distilled water were added to the mixture. After incubation for 90 min at room temperature, the absorbance was measured at 760 nm by using a multi-mode microplate reader (Spark, Tecan Group Ltd., Männedorf, Switzerland). The total phenolics contents were finally expressed as milligram of gallic acid equivalents per 100 g dry weight of *A. membranaceus* (mg GAE/100 g DW).

The total flavonoids content was measured by aluminium chloride colorimetric assay [18]. In brief, an aliquot (1 mL) of the extract or standard solutions of catechin (0, 20, 60, 100, 150, 200, 300, 400, 500, and 600 µg/mL) was added with 400 µL of 80% methanol and 50 µL of NaNO_2_ (5%, *w/v*) before incubation for 6 min. Afterward, 50 µL of AlCl_3_ (10%, *w/v*) were added. After another 6 min, 400 µL of NaOH (4%, *w/v*) were added to the mixture and further incubated for 15 min. The absorbance was measured at 510 nm. Catechin was chosen as the standard. The total flavonoids contents were expressed as milligrams of catechin equivalents per 100 g dry weight of *A. membranaceus* (mg CE/100 g DW).

### 2.4. UHPLC and UHPLC-MS/MS Analyses

UHPLC analysis was applied to isolate the bioactive compounds presented in *A. membranaceus*. A Thermo Scientific Ultimate 3000 UHPLC system coupled with an Orbitrap Elite (Thermo Fisher Scientific, Waltham, MA, USA) was used. A C18 column (3.0 × 100 mm, 1.8 μm, Agilent, Santa Clara, CA, USA) was equipped. The samples were eluted with a gradient system consisting of solvent A (0.1% formic acid in water, *v/v*) and solvent B (0.1% formic acid in acetonitrile, *v/v*). A gradient elution program was conducted as follows: 0–3 min, 3% solvent B; 3–24 min, from 3% to 95% solvent B; 24–29 min, 95% solvent B; 29–30 min, from 95% to 3% solvent B; 30–35 min, 3% solvent B. The flow rate was 0.4 ml/min and the injection volume was 2 µL. The column temperature was maintained at 35 °C. Electron spray ionization of the analytes was used in positive mode. Mass spectra were collected in the full-scan mode in a mass range of 100–1000 Da. The MS/MS spectra were obtained with collision energy of 25 eV.

### 2.5. Determination of Oxygen Radical Absorbance Capacity (ORAC)

The oxygen radical absorbance capacity assay was conducted by following the protocol of literature [19]. The fluorescein sodium salt and ABAP were dissolved in 75 mM PBS buffer (pH 7.4), respectively. A total 20 µL of sample solution or positive control or blank were added in 96-well microplate (Corning Scientific, Corning, NY, USA) and incubated for 30 min at 37 °C. Then, 200 µL of fluorescein sodium salt (0.96 µM) were added. After incubating for 20 min, 20 µL of ABAP (119 mM) were added to the mixture and measured immediately at 37 °C. The fluorescence generation was monitored every 1.5 min for 60 cycles by using a multi-mode microplate reader (excitation wavelength of 480 nm, and emission wavelength of 520 nm). The Trolox equiv (TE) solutions (6.25, 12.5, 25, and 50 μM) were used as the positive control, and 75 mM phosphate (pH 7.4) was used as the blank. ORAC values were expressed as µmol of Trolox equivalents per gram of *A. membranaceus* methyl extract (µmol TE/g) on the basis of the regression equation between Trolox concentration and the net area under the curve (AUC).

### 2.6. Determination of DPPH Radical Scavenging Activity

The DPPH radical scavenging activity of extract was evaluated by using the method described previously [20]. The DPPH in methanol (100 μM) was prepared freshly, and ascorbic acid (AA) was prepared as the positive control. A total 180 µL of DPPH in methanol were added into 20 µL of sample solutions and incubated for 30 min in dark at room temperature. Afterward, the absorbance was measured at 517 nm immediately. The vehicle control was set up by using methanol instead of the sample. The DPPH scavenging activity was calculated according to the following equation: Scavenging activity (%) = (1 − absorbance of sample/absorbance of control) × 100, and the IC_50_ value was calculated on the scavenging activity against DPPH radicals and expressed as µmol of ascorbic acid per gram of *A. membranaceus* extract (µmol AA/g).

### 2.7. Cytotoxicity Assay

HepG2 cells were obtained from the laboratory of Animal Center, Sun Yat-Sen University, China. The cells were cultured in William’s medium E (WME) supplemented with 5% FBS (Gibco Life Technologies, Grand Island, NY, USA), 50 units/mL penicillin, 2 mM glutamine, 100 μg/mL gentamicin, 10 mM Hepes, 50 μg/mL streptomycin, 5 μg/mL insulin, and 0.05 μg/mL hydrocortisone. Then the cell was cultured in a humidified incubator at 37 °C with 5% CO_2_.

The cytotoxicity assay was performed by the MTS staining method. Briefly, HepG2 cells were seeded in a 96-well microplate with a concentration of 4 × 10^4^ cells/well. After incubation for 24 h, the cells were collected by removing the growth medium from each well and were washed by PBS buffer. The sample with various concentrations (0, 25, 50, 100, 200 μg/mL) was used to treat the cells for 24 h. MTS solution (50 μg/mL) was added to each well and incubated for 4 h. The absorbance was measured at 570 nm.

### 2.8. Cellular Antioxidant Activity

The cellular antioxidant activity was analysed as previously reported [19]. Briefly, HepG2 cells were seeded at 6 × 10^4^ cells/well in a black 96-well microplate and inoculated at 37 °C for 24 h. The growth medium was then removed and each well was washed with 100 μL of PBS buffer. Afterward, the wells were treated with 100 μL of WME medium containing different concentrations of samples (0, 3.125, 6.25, 12.5, 25, 50 μg/mL) and 50 μM 2,7-diacetate dichlorofluorescein (DCFH-DA) were added to the cells and incubated for 1 h (Trolox at 3.125, 6.25, 12.5, 25, and 50 mM were used as control). The treatment solution was removed, and the 96-well microplate was washed three times with PBS, followed by the addition of an oxidant-supplemented medium (HBSS with 10 mM HEPES, 600 μM ABAP). The 96-well microplates were transferred to a multimode microplate reader. The fluorescence was recorded every 5 min for 1 h at 37 °C with an emission wavelength of 538 nm and excitation wavelength of 485 nm. After subtraction of the blank from each sample measurement, the area under the curve for fluorescence versus time was integrated to calculate CAA value as follows: CAA (units) = 1 − (∫SA/∫CA). In this equation, ∫SA is the integrated area under the fluorescence-time curve of samples, and ∫SA represents the integrated area under the curve of the control. By referring to the regression equation of concentration and CAA value, the EC_50_ values and quercetin equivalents were calculated.

### 2.9. Statistical Analysis

All the experiments were performed in triplicate and the results were expressed as mean ± standard deviation. The peak heights of chemicals in mass spectra were recorded and averaged over three replicates. Principal component analysis (PCA) was performed to obtain an overview of variations between data. Meanwhile, a supervised multivariate statistical method, orthogonal partial least square discriminant analysis (OPLS-DA) mode was built to find the analytes in the data with variable importance in the projection. Moreover, variable importance in projection (VIP) is an important index. The differential constituents were selected based on VIP values > 1 and *p*-values from Student’s *t*-test < 0.05 [21]. Heatmap was applied to visualize the number of differential metabolites and to analyse their hierarchical cluster. The results were subjected to analysis of variance and differences between means by using ANOVA test followed by Tukey’s test or Dunnett’s test. In all the tests, the differences between results were regarded as significant at *p* < 0.05.

## 3. Results

### 3.1. Total Phenolics and Flavonoids

**Figure 1:**
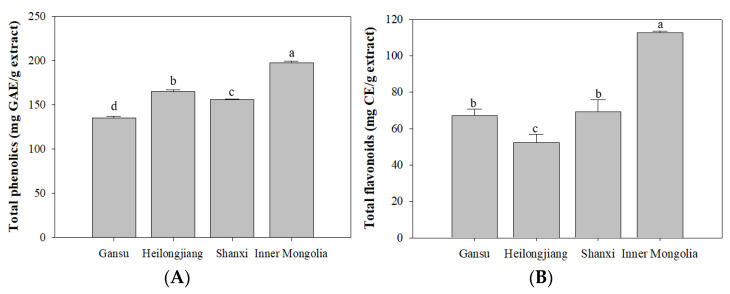
Total phenolics and total flavonoids contents of *A. membranaceus* roots from different origins (Gansu, Inner Mongolia, Shanxi, and Heilongjiang). (**A**), total phenolics contents; (**B**), total flavonoids contents. Values in one column with different letters are significantly different (*p* < 0.05).

The yield of *A. membranaceus* extract were 17.11% (Gansu), 16.84% (Shanxi), 14.84% (Heilongjiang), and 10.13% (Inner Mongolia), respectively. Total phenolics and flavonoids contents of *A. membranaceus* root from four origins are presented in Figure 1. The content of phenolics varied between 135.23–197.40 mg GAE/g extract and in a decreasing order of ‘Inner Mongolia’ (197.40 ± 1.95 mg GAE/g extract), ‘Heilongjiang’ (164.93 ± 2.14 mg GAE/g extract), ‘Shanxi’ (155.62 ± 0.95 mg GAE/g extract), and ‘Gansu’ (135.24 ± 1.66 mg GAE/g extract), respectively. Among them, the ‘Inner Mongolia’ and ‘Heilongjiang’ samples presented significantly (*p* < 0.05) higher levels of phenolics than the other two origins. Meanwhile, the total flavonoids contents of *A. membranaceus* were significantly different among the four origins. It ranged from 52.27 to 112.75 mg CE/g extract. Among them, the ‘Inner Mongolia’ samples (112.75 ± 0.77 mg CE/g extract) presented significantly higher (*p* < 0.05) flavonoids contents than the other three origins, followed by ‘Gansu’ (67.22 ± 3.67 mg CE/g extract) and ‘Shanxi’ (69.12 ± 6.59 mg CE/g extract) samples. Unlike the trend of phenolic compounds, a relatively low flavonoids content was observed in ‘Heilongjiang’ sample (52.27 ± 4.63 mg CE/g extract), which indicated the presence of non-flavonoids phenolics.

It is widely known that a diet rich in fruit and vegetables has a protective effect against cancer insurgence and development. In the presence of an intense stressing event, cells activate specific responses to counteract cell death or senescence, which is known to act as a key task in the onset of age-related pathologies and the loss of tissue homeostasis [22]. Phenolics are generally recognized to be responsible for antioxidant and anti-aging effects, as well other beneficial actions [23]. Previous research showed that the total phenolic content of *A. membranaceus* from Western Siberia ranged from 100–190 mg GAE/g extract [24], which is consistent with this study. The total phenolics and flavonoids contents of *A. membranaceus* from Shijiazhuang origin were 27.646 ± 0.11 mg GAE/g extract and 7.048 ± 0.87 mg RE/g extract, respectively [25]. The difference of total phenolics and flavonoids contents distinguished the *A. membranaceus* materials from different origins clearly.

### 3.2. Identification of Bioactive Compounds

**Figure 2:**
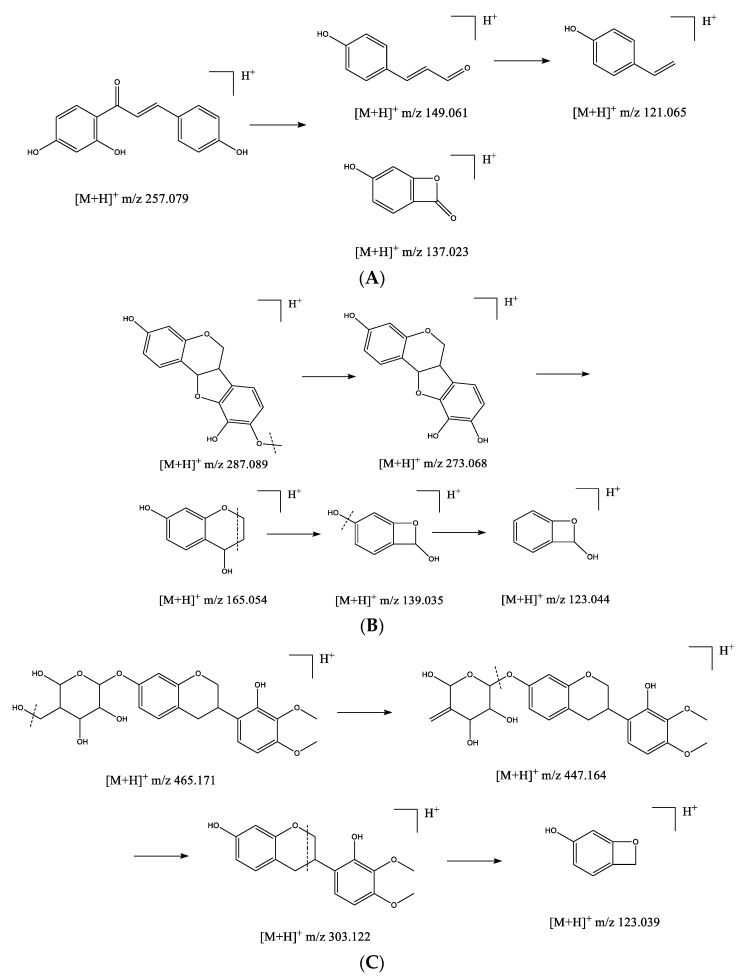
The putative fragmentation pathways of isoliquiritigenin (**A**), vesticarpan (**B**), and isomucronulatol 7-*O*-glucoside (**C**).

The UHPLC–MS/MS analysis of the methanolic extract was performed to characterize the bioactive compounds of *A. membranaceus.* The total ion chromatogram was presented in positive ion mode. After preliminary comparative analysis by retention time, MS/MS fragments, and the reported data in references or Compound Discoverer, 34 flavonoids were identified, including 13 methoxylated flavonoids, 15 flavonoid glycosides, and 6 flavonols. The compounds identified by UHPLC-MS/MS spectra are summarized in Table 1, and their putative fragmentation pathways are shown in Figure 2. The identification of methoxylated flavonoids and flavonoids glycosides were explained in detail as follows.

#### 3.2.1. Polymethoxylated Flavonoids

Polymethoxylated flavonoids are characteristic compounds distributed in *Astragalus* species. In positive ion mode, the main feature of the fragmentation of [M+H]^+^ ions of methoxylated flavonoids is the loss of methyl radical (form the fragment ions of [M+H-nCH_3_]^+^). Besides, neutral loss could be observed, such as 28 (CO) and 44 (CO_2_). The C ring of flavonoids was less stable and prone to be cleaved resulting in various retro-Diels Alder fragments [26]. The even-electron rule could be applied for the identification of methoxylated flavonoids [27]. For example, compound **29** with the retention time of 15.87 min was identified as formononetin due to the quasi-molecular ion peak at *m*/*z* 269.077 ([M+H]^+^). The characteristic MS/MS ion peak at *m*/*z* 254.056 ([M+H-CH_3_]^+^) was detected. A dominant fragment ion ^1,3^A^+^ at *m*/*z* 137.022 was presented due to the breakage of C ring. Similarly, vesticarpan (22) at the retention time of 14.37 min had a quasi-molecular ion at *m*/*z* 287.089 ([M+H]^+^). The fragment at 273.068 matched the loss of a methyl residue ([M+H-CH_3_]^+^) and yielded secondary fragment ions at *m*/*z* 165.054 (^6,7^A^+^) and 139.035 (^1,3^A^+^). Furthermore, the fragment ion at *m*/*z* 123.044 was observed due to the loss of 18 (H_2_O). The putative fragmentation pathway is shown in Figure 2. Methylinissolin (**20**) at the retention time of 13.70 min had a base peak at *m*/*z* 315.201 ([M+H]^+^). The typical fragment at *m*/*z* 300.062 was detected due to the loss of methyl radical and yielded secondary fragment ions ^1,3^A^+^ at *m*/*z* 123.044. The A-ring and/or B-ring were easy to produce fragment ions due to the neutral loss of CH_4_. The peaks with the retention times of 13.10, 13.14, 13.47, 13.66, 13.78, 14.81, 15.65, 15.66, and 16.09 min were assigned to be methoxylated flavonoids with [M+H]^+^ at *m*/*z* 301.103, 303.118, 331.078, 285.071, 315.084, 317.099, 301.069, 271.086, and 299.088, respectively. These fragments were characterized by the losses of methyl radicals and fragment ^1,3^A^+^ was the most abundant ion in all the spectra. These compounds were identified as 7-hydroxy-2′-methoxy-4′,5′-methylenedioxyisoflavane (**14**), 2′,8-dihydroxy-4′,7′-dimethoxyisoflavane (**16**), chrysin (**17**), calycosin (**19**), odoratin (**21**), astragaluquinone (**24**), pratensein (**26**), pinostrobin (**27**), and 7-hydroxy-3′,5′-dimethoxyisoflavone (**31**). Additionally, compounds **25** and **32** had the quasi-molecular ion peaks at *m*/*z* 257.079 and 273.183 were identified as isoliquiritigenin (**25**) and butein (**32**), two principle chalcones, which further formed secondary fragments at *m*/*z* 149.061, 137.023, 121.065, and 165.091, 137.059, consistent with literature [28]. These were the characteristic fragment ions produced by chalcone fragmentation pathway (Figure 2A).

#### 3.2.2. Flavonoid Glycosides

Flavonoid glycosides, including flavonoid *C*-glycosides and *O*-glycosides, were two common patterns distributed in plants with multiple bioactivities [29]. In this study, 15 flavonoid *O*-glycosides were found in *A. membranaceus*, while 12 of them were identified to bound one or two methyls. After comparing the quasi-molecular ions and fragment ions in MS/MS spectra with those of previously reported literature [30,31], they were identified as narcissin (**1**), flagaloside D (**3**), licoagroside D (**5**), calycosin 7-*O*-glucopyranoside (**6**), odoratin 7-*O*-glucopyranoside (**7**), biochanin A 7-*O*-(6-*O*-malonyl-glucoside) (**9**), pratensein 7-O-glucopyranoside (**10**), ononin (**11**), methylinissolin 3-*O*-glucoside (**12**), isomucronulatol 7-*O*-glucoside (**13**), respectively. As reported previously, the application of low or medium fragmentation energy results in heterolytic cleavage of their hemiacetal C-O bonds [32]. Besides, the losses of 18 (H_2_O), 28 (CO), 42 (C_2_H_2_O), 44 (CO_2_) are also characteristic for flavonoid glycoside [33]. In the present work, flavonoid *O*-glycosides showed similar fragmentation pattern. For instance, compound **13** with the retention time of 13.06 min was identified as isomucronulatol 7-*O*-glucoside due to the quasi-molecular ion peak at *m*/*z* 465.17 ([M+H]^+^) and the characteristic MS/MS ion at *m*/*z* 447.16 ([M+H-H_2_O]^+^), and ion at *m*/*z* 303.12 ([M-C_6_H_10_O_5_]^+^) corresponding to the loss of a glucose moiety. The possible fragmentation pattern is proposed in Figure 2C. Furthermore, Compound **11**, with parent ion at *m*/*z* 412.54, was identified as ononin (C_22_H_22_O_9_), which fragmented into daughter ion at *m*/*z* 269.08 due to the loss of a glucose moiety and further yielded the ion at *m*/*z* 254.06 ([M-C_6_H_10_O_5_-CH_3_]^+^), consistent with the reported data [34]. Compound **6,** with the base peak at *m*/*z* 447.124 ([M+H]^+^), matched the ion [Y_0_]^+^ at *m*/*z* 285.074 with the loss of a glucose residue. The presence of fragment at *m*/*z* 270.051 was due to the loss of a methyl radical [Y_0_-CH_3_]^+^. Besides, methylinissolin 3-*O*-glucoside (**12**) at the retention time of 12.88 min had a quasi-molecular ion at *m*/*z* 463.155 ([M+H]^+^). The fragments at *m*/*z* 445.149 and 301.105 were attributed to [M+H-H_2_O]^+^ and [Y_0_+H]^+^, respectively. The ion ^6,7^A^+^at *m*/*z* 165.054 was the dominant fragment ion due to the breakage of C ring. Moreover, the compounds **2**, **4**, and **8** with the retention times at 9.41, 10.58, and 11.21 min were identified as nicotiflorin, liquiritin, and apigenin 7-*O*-glucopyranoside with the same fragmentation pattern.

#### 3.2.3. Multivariate Statistical Analysis

**Figure 3:**
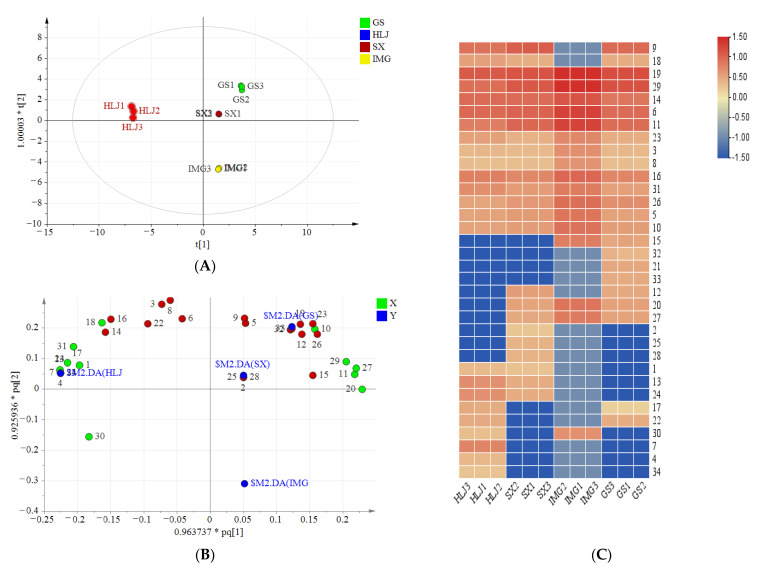
Multivariate statistical analysis of flavonoids in *A. membranaceus* from Gansu (GS), Heilongjiang (HLJ), Shanxi (SX), and Inner Mongolia (IMG): (**A**) OPLS-DA score plot; (**B**) OPLS-DA loading plot. The important compounds are in red colour. (**C**) Heatmap of phenolic components in astragalus from different origins. The levels of identified compounds were compared by their peak integration.

PCA analysis is an unsupervised method usually employed to determine patterns between multivariate samples. The PCA analysis showed a clear tendency of separation among *A. membranaceus* samples from four origins. Specifically, the first two principal components explained 77.9% of the total variance, in which the first principal component explained 51.2% and the second principal component explained 26.7%. The first principal component was represented by the compounds such as nicotiflorin, liquiritin, ononin, 7-hydroxy-2′-methoxy-4′,5′-methylenedioxyisoflavane, calycosin 7-*O*-{6″-[-but-2-enoyl]}-glucoside, methylinissolin, isoliquiritigenin, daidzein, and astragaisoflavane D. Most of them presented higher content in the ‘Inner Mongolia’ extract. The supervised discriminant OPLS-DA was performed to classify the samples from four regions and find out the differential compounds. A model with R^2^X of 0.997, R^2^Y of 0.998, and Q^2^ of 0.99 was constructed. R^2^Y > 0.9 indicated an excellent fitted model, and Q2 > 0.9 suggested a good repeatability and predictability of the model. As shown in Figure 3A, no serious outlier was observed. The outlier is the plot out of the ellipse, which is defined as the Hotelling’s T2 range 95% confidence [35]. The samples from different origins exhibited good separation. The ‘Heilongjiang’ sample and ‘Inner Mongolia’ sample were separated significantly in t [1] direction. ‘Gansu’ sample and ‘Shanxi’ sample located near the centre of the model plane and stayed close to each other, which indicated that their chemical compositions were similar. According to the searching rule of VIP value > 1 and *p*-value < 0.05, a total of 18 phenolics were regarded as principal metabolites marked in red in Figure 3B. Six methoxylated flavonoids (compounds 7-hydroxy-2′-methoxy-4′,5′-methylenedioxyisoflavane, 2′, 8-Dihydroxy-4′, 7-dimethoxyisoflavane, calycosin, vesticarpan, pratensein, 3′, 6-dihydroxy-4′-methoxy-aurone), eight flavonoid glycosides (compounds flagaloside D, licoagroside D, calycosin 7-*O*-glucoside, biochanin A 7-*O*-(6-*O*-malonyl-glucoside), methylinissolin 3-*O*-glucoside, calycosin 7-*O*-{6″-[-but-2-enoyl]}-glucoside, nicotiflorin, apigenin 7-*O*-glucoside, while the first six of them having one or two methyl groups), and four flavonols (apigenin, isoliquiritigenin, daidzein, butein) were included. Different geographical locations led to the variation of metabolites accumulation.

The heat map was applied to demonstrate the variation of the identified compounds. As shown in Figure 3C, ‘Inner Mongolia’ extract was rich in flavonoid glycosides and methoxylated flavonoids. Most principal flavonoids including flagaloside D, licoagroside D, calycosin 7-*O*-glucoside, calycosin 7-*O*-{6″-[-but-2-enoyl]}-glucoside, apigenin 7-*O*-glucoside, 7-hydroxy-2′-methoxy-4′,5′-methylenedioxyisoflavane, 2′,8-dihydroxy-4′,7-dimethoxyisoflavane, calycosin, and pratensein all presented high levels in ‘Inner Mongolia’ samples than others. Besides, the principal methoxylated flavonoids such as vesticarpan and 6,3′-dihydroxy-4′-methoxy-auron were higher in the extract of ‘Gansu’ and ‘Heilongjiang’ samples than ‘Shanxi’ samples. Conversely, two principal flavonols (isoliquiritigenin and daidzein), only existed in ‘Shanxi’ sample. It could be used to distinguish ‘Gansu’ and ‘Shanxi’ samples. To compare the chemicals of ‘Heilongjiang’ with the other origins, the flavonoid glycosides and methoxylated flavonoids, such as flagaloside D, calycosin 7-*O*-glucopyranoside, apigenin 7-*O*-glucopyranoside, 7-hydroxy-2′-methoxy-4′,5′-methylenedioxyisoflavane, and 8,2′-dihydroxy-7,4′-dimethoxyisoflavane, were presented at relatively lower contents, which were defined as the characteristic compounds of the ‘Heilongjiang’ sample.

### 3.3. Antioxidant Activity

#### 3.3.1. ORAC Value and DPPH Radical Scavenging Activity

**Figure 4:**
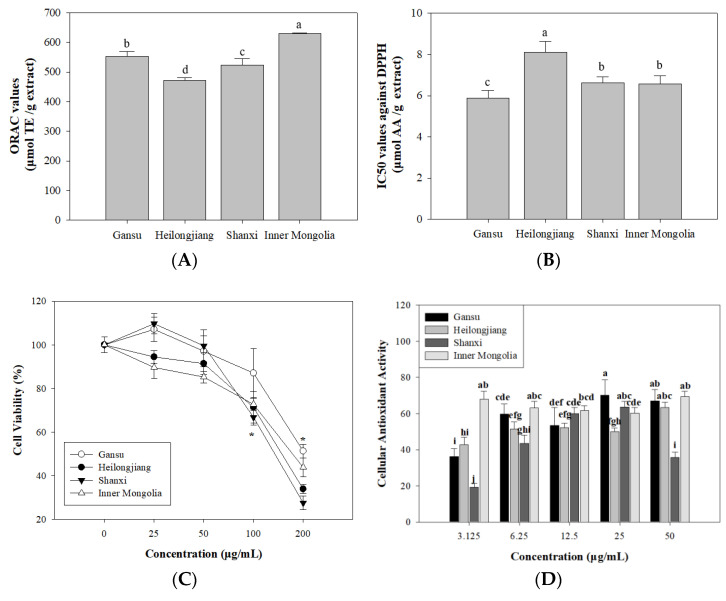
Antioxidant activities of *Astragalus* extracts from different origins. (**A**), ORAC value; (**B**), DPPH radical scavenging activity; (**C**), Cytotoxicity; (**D**), Cellular antioxidant activity. Different letters indicate values have significant differences (*p* < 0.05).

ORAC assay is a valid method to evaluate the antioxidant ability through monitoring the inhibition capacity against peroxyl radical [36]. The breakdown of ABAP can provide peroxyl radical and leads to subsequent oxidation. It can be monitored through fluorescent intensity change. As shown in Figure 4A, ‘Inner Mongolia’ (628.94 ± 3.30 μmol TE/g extract) showed significant (*p* < 0.05) higher ORAC values. The ORAC values of other three samples were in a decreasing order of ‘Gansu’ (553.18 ± 15.28 μmol TE/g extract), ‘Shanxi’ (522.48 ± 21.91 μmol TE/g extract), and ‘Heilongjiang’ (471.29 ± 8.61 μmol TE/g extract). It indicated that ‘Inner Mongolia’ and ‘Gansu’ extracts processed good peroxyl radical inhibition activities.

DPPH test is widely used to evaluate the antioxidant capacity of phenolics [37]. It is based on a stable nitrogen-centred free radical that is characterized by absorbance at 517 nm with a deep violet colour. In the presence of free radical scavenger, the absorbance of DPPH will decrease due to hydrogen donation from antioxidant with a dose-dependent behaviour [38]. As shown in Figure 4B, ‘Heilongjiang’ extract presented a higher DPPH radical scavenging activity with an IC_50_ value of 8.10 ± 0.54 μmol AA/g extract, followed by ‘Shanxi’ extract (IC_50_ of 6.63 ± 0.30 μmol AA/g extract) and ‘Inner Mongolia’ extract (IC_50_ of 6.57 ± 0.40 μmol AA/g extract). The ‘Gansu’ extract possessed the lowest IC_50_ value of 5.89 ± 0.36 μmol AA/g extract. This order was inconsistent with that of ORAC assay.

The occurrence of oxidation process is correlated with the existence of a surplus of free radicals, which are responsible for multiple diseases [39]. Peroxyl radicals are characterized as free radicals that predominate in the lipid oxidation of biological system [40]. Its inhibition plays an important role in disease prevention. Generally, the radical-trapping antioxidant activity of flavonoids is related to the hydrogen atom transfer to a peroxyl radical [41]. It can be used to explain the different radical-inhibitory activities of four origins samples. *A. membranaceus* from Lebanon possesses the lowest IC50 value of 102 ± 4.4 μg/mL [42]. Several bioactive compounds have been confirmed. For example, formononetin, calycosin, and calycosin-7-*O*-glucoside showed superior antioxidant activity and inhibited free radicals generated by DPPH in a dose-dependent manner [43]. Moreover, calycosin can enhance antioxidant enzymatic activities such as glutathione peroxidase, catalase, superoxide dismutase and attenuate H_2_O_2_-induced H_9_C_2_ cell apoptosis rate in a dose-dependent manner as well [44]. Besides, the chemicals of formononetin from *A. membranaceus* have evidenced the capacity of inhibiting xanthine oxidase-induced cell injury significantly [45]. They were the major flavonoids in *A. membranaceus*.

#### 3.3.2. Cellular Antioxidant Activity (CAA)

HepG2 cells line is a sensitive cell model in the determination of antioxidant biomarkers [46]. In the present work, the cytotoxic effects of *A. membranaceus* extract at different levels (25, 50, 100, and 200 μg/mL) against HepG2 cells were determined by MTS assay. From the results summarized in Figure 4C, no significant cytotoxicities were observed between extract-treated cells and untreated cells within concentration of 0–50 μg/mL. It indicated that this range could be used for cellular antioxidant activity assay.

The cellular antioxidant activities of *A. membranaceus* extracts were evaluated by CAA assay, and the results are shown in Figure 4D. It was obvious that the *A. membranaceus* extracts from four origins could protect HepG2 cells against peroxyl radicals with a dose-dependent effect. Among them, ‘Inner Mongolia’ sample showed a higher CAA value than the others at 3.125 μg/mL, while ‘Gansu’ and ‘Shanxi’ extracts exhibited higher CAA values at 25 μg/mL. Trolox was used as positive control.

CAA assay is performed based on polarity, solubility, and molecular weight of the antioxidant and provides an important tool for the biological assessment of antioxidant activity [47]. In the CAA assay, the DCFH-DA is preloaded into the cell, treated with the intracellular peroxyl radicals generated from ABAP and therefore the fluorescence level is recorded [48]. Nuclear transcription factor, erythroid 2-like 2 (Nrf2) is a central conditioner of antioxidant response elements [49]. Phenolic compounds can protect HepG_2_ cell against oxidative injury by promoting the Nrf2 translocation, which subsequently attenuates oxidative DNA damage, induce the expression of antioxidant enzymes, and reduce cellular ROS formulation [50,51,52]. The antioxidant activities of phenolics usually depend on the chemical construction of attached functional groups and their permutation. Previous research has mentioned that methoxyl and hydroxyl groups are directly related to the radical-inhibited ability [53]. When the same skeleton was presented, the presence of methoxyl usually brings an enhanced antioxidant activity for phenolics. However, sometimes this substitution by methoxyl can diminish the antioxidant activity [54]. The substitution pattern on the B-ring is important to the antioxidant activity of flavonoids.

## 4. Conclusions

As mentioned above, thirty-four flavonoids in *A. membranaceus* from different origins were identified by UHPLC-MS/MS. Eighteen identified compounds were considered to be significantly important based on OPLS-DA analysis. It characterized the samples from different origins, especially for ‘Heilongjiang’ and ‘Inner Mongolia’. Through antioxidant activity evaluation, ‘Inner Mongolia’ extract presented significantly higher ORAC and CAA values (*p* < 0.05). It was consistent with its higher phenolics and flavonoids contents. Further investigation on in vivo antioxidant mechanisms of those flavonoids is worthy to be conducted in the future.

## Figures and Tables

**Table 1:** The identified flavonoids compounds of Astragalus based on the UHPLC-ESI-MS/MS method.

NO.	RT (Min)	Chemicals	[M+H]^+^	Formula	MS2 Fragments (*m*/*z*)
1	8.64	Narcissin	625.172	C_28_H_32_O_16_	463.121
2	9.41	Nicotiflorin	595.161	C_27_H_30_O_15_	433.111, 271.059
3	9.58	Flagaloside D	581.196	C_26_H_28_O_15_	563.183, 419.143
4	10.58	Liquiritin	419.129	C_21_H_22_O_9_	257.071
5	10.77	Licoagroside D	449.140	C_22_H_24_O_10_	287.081
6	10.88	Calycosin 7-*O*-glucoside	447.124	C_22_H_22_O_10_	285.074, 270.051
7	10.98	Odoratin 7-*O*-glucoside	477.135	C_23_H_24_O_11_	315.085, 300.062
8	11.21	Apigenin 7-*O*-glucoside	433.108	C_21_H_20_O_10_	401.119, 271.059
9	11.67	Biochanin A 7-*O*-(6-*O*-malonyl-glucoside)	533.124	C_25_H_24_O_13_	489.137, 447.126, 285.075, 270.052
10	11.78	Pratensein 7-*O*-glucoside	463.119	C_22_H_22_O_11_	301.069, 286.050
11	12.54	Ononin	431.130	C_22_H_22_O_9_	269.079, 254.057
12	12.88	Methylinissolin 3-*O*-glucoside	463.155	C_23_H_26_O_10_	445.149,301.105, 165.054
13	13.06	Isomucronulatol 7-*O*-glucoside	465.171	C_23_H_28_O_10_	447.164, 303.122, 275.090
14	13.10	7-Hydroxy-2′-methoxy-4′,5′-methylenedioxyisoflavane	301.103	C_17_H_16_O_5_	286.083, 270.087, 123.044
15	13.10	Calycosin 7-*O*–{6″-[-but-2-enoyl]}-glucoside	515.150	C_26_H_26_O_11_	429.152, 285.075, 270.050
16	13.14	2′, 8-Dihydroxy-4′, 7-dimethoxyisoflavane	303.118	C_17_H_18_O_5_	275.091, 153.054, 123.044
17	13.47	Chrysin	331.078	C_17_H_14_O_7_	316.056, 137.029
18	13.60	Kaempferol	287.090	C_15_H_10_O_6_	271.054, 153.054, 137.023,
19	13.66	Calycosin	285.071	C_16_H_12_O_5_	270.051, 137.023
20	13.70	Methylinissolin	315.201	C_18_H_18_O_5_	300.062, 165.054
21	13.78	Odoratin	315.084	C_17_H_14_O_6_	287.089, 137.059
22	14.37	Vesticarpan	287.089	C_16_H_14_O_5_	272.068, 165.054, 123.044
23	14.52	Apigenin	271.059	C_15_H_10_O_5_	153.017, 137.019
24	14.81	Astragaluquinone	317.099	C_17_H_16_O_6_	302.073, 123.044
25	15.44	Isoliquiritigenin	257.079	C_15_H_12_O_4_	149.061, 137.023, 121.065
26	15.65	Pratensein	301.069	C_16_H_12_O_6_	286.046, 153.018
27	15.66	Pinostrobin	271.086	C_16_H_14_O_4_	256.062, 137.059
28	15.84	Daidzein	255.099	C_15_H_10_O_4_	137.059, 119.044
29	15.87	Formonentin	269.077	C_16_H_12_O_4_	254.056, 137.022
30	16.06	Garbanzol	273.183	C_15_H_12_O_5_	255.173, 137.059
31	16.09	7-Hydroxy-3′,5′-dimethoxyisoflavone	299.088	C_17_H_14_O_5_	284.067, 271.058, 137.058
32	16.65	Butein	273.183	C_15_H_12_O_5_	165.091, 137.059
33	17.81	3′,6-Dihydroxy-4′-methoxy-aurone	285.073	C_16_ H_12_ O_5_	285.075, 270.051, 121.027,
34	18.49	Astragaisoflavane D	603.218	C_34_H_34_O_10_	287.091, 272.068

## Data Availability

All data presented are available in the manuscript.
